# Accurate Pupil Center Detection in Off-the-Shelf Eye Tracking Systems Using Convolutional Neural Networks

**DOI:** 10.3390/s21206847

**Published:** 2021-10-15

**Authors:** Andoni Larumbe-Bergera, Gonzalo Garde, Sonia Porta, Rafael Cabeza, Arantxa Villanueva

**Affiliations:** Arrosadia Campus, Public University of Navarre, 31006 Pamplona, Spain; gonzalo.garde@unavarra.es (G.G.); sporta@unavarra.es (S.P.); rcabeza@unavarra.es (R.C.); avilla@unavarra.es (A.V.)

**Keywords:** eye tracking, pupil center detection, convolutional neural networks

## Abstract

Remote eye tracking technology has suffered an increasing growth in recent years due to its applicability in many research areas. In this paper, a video-oculography method based on convolutional neural networks (CNNs) for pupil center detection over webcam images is proposed. As the first contribution of this work and in order to train the model, a pupil center manual labeling procedure of a facial landmark dataset has been performed. The model has been tested over both real and synthetic databases and outperforms state-of-the-art methods, achieving pupil center estimation errors below the size of a constricted pupil in more than 95% of the images, while reducing computing time by a 8 factor. Results show the importance of use high quality training data and well-known architectures to achieve an outstanding performance.

## 1. Introduction

Eye tracking technology appeared on the 20th century with the purpose to detect eye position and to follow eye movements. Recently, eye tracking technology has suffered an increasing growth due to its use on virtual reality (VR) and augmented reality (AR) devices, as well as its multiple applications for many research areas as gaze estimation [1,2], human computer interaction (HCI) [3], assistive technologies [4], driving assistance systems [5], biometrics [6], or psychology and marketing analysis [7,8,9] among others.

As in many other areas, one of the goals of eye tracking techniques is to be less invasive. Early eye tracking methods as the scleral coil or electro-oculography (EOG) involved to use contact lenses with coils of wire attached to them or to arrange several electrodes around the users’ eyes [10]. Afterward, less invasive systems such as infrared oculography (IRO) or video-oculography (VOG) appeared. IRO methods are based on an infrared emitter that radiates certain amount of light which is reflected in the eye and detected by an infrared detector. VOG methods are based on the use of cameras and image processing. Eye position detection and eye movements tracking are performed by detecting specific features related to the shape or appearance of the eye, being the pupil center one of the most important features [11].

The use of IRO and VOG methods in controlled environments where the user is using head mounted devices or in which the movement of the user is limited has allowed to develop highly accurate systems [12]. Feature and model-based eye tracking systems have demonstrated to be simpler and more accurate approaches and have become the consensus solution [1,13]. Works applying machine learning techniques for semantic segmentation [14,15,16] or pupil center detection [17,18] in these controlled environments can be found. The use of convolutional neural networks (CNN) has proven to be a robust solution for pupil center detection methods in challenging images with artifacts due to poor illumination, reflections or pupil occlusion [17,18].

**Figure 1:**
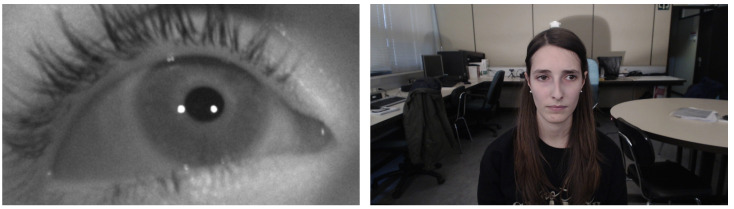
**Left**: image captured by a system with high focal length cameras and using infrared light [19]. **Right**: image captured by using a webcam [20].

In recent years, even less invasive systems have been developed. In those systems users do not have to carry any device and their movement is not limited. However, several new problems appeared and the well-known theories and models about head mounted VOG and infrared eye tracking cannot be directly applied. More precisely, IRO-based methods cannot be used due to the fact that the light emitter cannot be kept oriented towards the eye. Regarding VOG-based methods, eye tracking must be done using cameras with a lower focal length (e.g., webcams) and without using infrared light. In addition, the fact of using shorter focal lengths reduces the resolution of the ocular zone. Figure 1 shows the differences between images captured by systems using infrared light and high focal lengths (left) and images obtained by using a commercial webcam (right). It can be noted that in the left image, the user’s movement is restricted due to the high focal length of the camera and the infrared lights, while, in the right one, the user can move more freely.

For this non-invasive webcam scenario, the relevance of learning and training methodologies begins to be more important and shows up as a promising tool [21,22,23]. These training-based techniques require a large amount of images representing the variability of the problem in order to get adapted to the solution and be able to generalize. Thus, availability of properly annotated databases is one of the cornerstones of the success of any deep or machine learning technique. In the case of pupil center estimation, databases containing eye landmarks are essential. Although several datasets in which key face landmarks are provided as labels can be found in the literature for face detection purposes, there are no many datasets containing accurate pupil center annotations.

In this work, a pupil center manual labeling procedure of a well-known face landmark dataset has been made, resulting on a novel database named Pupil-PIE (PUPPIE) containing a total of 1791 annotated images and representing the first contribution of this work. The second contribution is a method based on convolutional neural networks (CNNs) for pupil center detection over webcam images. The main idea is not to create a method based on a new and complicated architecture but to see if, with a well-known architecture and sufficiently good training data, it is possible to obtain results that outperform the state-of-the-art. For that purpose, a model based on a ResNet-50 [24] architecture has been trained to compute the x and y coordinates of the pupil center in the image using PUPPIE dataset. The model has been tested using both real and synthetic state-of-the-art databases.

The paper is organized as follows. In next section, a brief review of the state-of-the art is made. In Section 3, the databases used in this work, as well as the algorithm employed for pupil center detection are presented. The explanation of the metrics used and the experiments carried out along with the evaluation of the results obtained from the method are done in Section 4. Finally, in Section 5, the conclusions of the work are summarized.

## 2. Related Works

As it has already been said, in high resolution scenarios feature and model-based eye tracking systems have demonstrated to be simpler and more accurate approaches becoming the consensus solution. However, when moving to lower resolution systems, the freedom of movement of the user, as well as the large number of possible illuminations, focal lengths, or viewing angles, because that the well-known methods used in high resolution do not produce sufficiently accurate results, so new methods have to be developed for this scenario.

One of the first works regarding pupil center detection in low resolution images is the one presented by Valenti et al. [25] in which isophote curves, i.e., curves of equal intensity are used. In the work by Zhang et al. [26] isophotes are also used, and gradient features are employed to estimate pupil center locations. The isophote curves are calculated assuming that the large contrast in the pupil or iris area permits a rough estimation of the center by using a voting procedure. Additional stages as selective oriented gradient filter, energy maps post processing and iris radius constraints are required in order to achieve more accurate detection. Gradient information is also employed in the work by Timm and Barth [27]. They propose a mathematical function that reaches its maximum at the center of a circular pattern, which is the location where most of the image gradients intersect. Image topography and curve extraction is also employed by Villanueva et al. [28]. Skodras et al. [29] propose a method based on the use of color and radial symmetry. George and Routray [30] propose a two-stage algorithm which uses geometrical characteristics of the eye for iris center localization. First, a coarse location of the pupil center is obtained using a convolution-based approach derived from a circular Hough transform. Then, the pupil center location is refined using boundary tracing and ellipse fitting. Xiao et al. [31] propose a multi-stage method for real-time pupil center detection based on the combination of snakuscule [32], circle fitting, and binary connected component.

Pursuing in the area of machine learning techniques, cascaded regressors methods have demonstrated to be highly accurate and robust in facial landmark tracking [33,34]. In this manner, works applying cascaded regressors for pupil center detection and eye tracking can be found in recent publications. Larumbe et al. [35] propose a cascaded regressor based on supervised descent method [33] and random cascaded-regression copse [34] to detect the pupil centers. They use the histogram of oriented gradients (HOG) to perform the feature extraction. Gou et al. [36] propose a similar cascaded regression strategy but using Scale-invariant feature transform (SIFT) for feature extraction and trying to use eye synthetic images in order to augment the training data.

Another learning method which has demonstrated a great performance is regression trees (RT) technique [37]. In the work by Markuš et al. [38] a pupil localization method based on an ensemble of randomized regression trees is proposed. Kacete et al. [39] also use RT-based models to estimate head pose and 2D pupil center. In [40], Levin et al. propose a two-stage method for eye center detection based on cascaded regression trees and employing gradient histogram features. A circle fitting post-processing step is used in order to refine the regressor estimation.

Over the last decade, deep neural networks have proven to be a powerful tool in many areas of computer vision, such as image classification [24,41], object detection [42,43] or object segmentation [44,45], among many others. Therefore, in recent years, methods based on this technology have appeared to solve the problem of pupil detection. In the work made by Xia et al. [22], fully convolutional networks (FCN) are used to segment the pupil region. FCN is an end-to-end and pixels-to-pixels network used for segmentation tasks. The idea is to consider the pupil center localization as a semantic segmentation task and to design an FCN with a shallow structure and a large kernel convolutional block to locate the eye center. In the work made by Choi et al. [21], a FCN is also used to perform a pupil region segmentation. Additionally, a CNN is used to determine if the user is wearing glasses. If glasses are present, they are removed through CycleGAN [46]. Once the image is segmented, the pixel with the maximum intensity is determined as the pupil center. Another method robust against glasses wearing is the one proposed by Lee et al. [23]. That consists of an appearance-based pupil center detection, inspired by [21] but employing perceptual loss to mitigate the blur phenomenon produced by the glass removal network, and mutual information maximization to enhance the representation quality of the segmentation network. An additional objective is to reduce the computational time of the face detector and the glasses removal network by using non-local and self-attention blocks. Another work that employs a generative adversarial framework is the one proposed by Poulopoulos et al. [47]. They reformulate the eye localization problem into an image-to-heatmap regression problem and try to solve it in an unsupervised way. The architecture that they propose is composed by an encoder-decoder translator which transform the input images to heatmaps trained jointly with a discriminator that tries to distinguish the translated heatmaps from real ones. In [48], Kitazumi and Nakazawa propose a CNN-based pupil segmentation method which also consists of an encoder-decoder architecture for pupil segmentation and pupil center detection. They first perform an eye region detection using dlib [49] and use a five-layer U-Net [50] architecture for the pupil segmentation. Recently, Zdarsky et al. [51] proposed a method for gaze estimation with outstanding results. In the first stage of this method a facial landmark estimation is performed using DeepLabCut [52], an open-source toolbox for pose estimation of body parts based on deep-learning. DeepLabCut employs the feature detectors subset of DeeperCut [53] which consists of a pre-trained ResNet-50 [24] followed by deconvolution layers used to up-sample the visual information and produce spatial probability densities. The deconvolution layers are specific to each body part and its probability density represents the ’evidence’ that this specific body part is in a particular region [52]. However, the work of Zdarsky et al. does not include the accuracy of the estimated landmarks.

Some other works try to allow real-time pupil center detection without the need of using a GPU. In [54], Kim et al. use a cascade deep regression forest instead of a deep neural network. The objective is to design a more transparent and adoptable lightweight pupil tracking model reducing the number of parameters and operations. This method allows precise real-time pupil center detection using only a CPU. Cai et al. [55] propose a low computational cost method based on hierarchical adaptive convolution to localize the pupil center. They design different hierarchical kernels with which convolute the eye images. The kernel used for each image is selected using the 3D head pose of the user obtained by a previous localization stage.

## 3. Materials and Methods

### 3.1. Datasets

As already mentioned, in order to train a CNN-based model, a manual labeling procedure of the pupil centers in some facial landmark databases has been made. For testing the model, GI4E [28] dataset, I2Head [20] dataset, a subset of the MPIIGaze [56] dataset, and the U2Eyes [57] synthetic dataset have been used.

Table 1 summarizes the number of images on the original datasets, as well as the number of re-labeled images used in this work.

#### 3.1.1. PUPPIE

**Figure 2:**
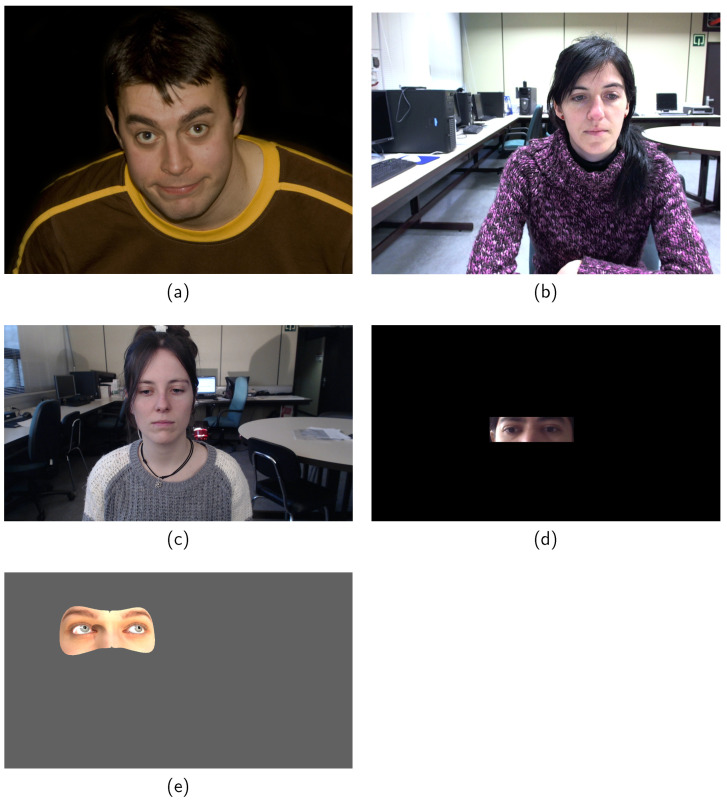
Images from the datasets used in this work. (**a**) HELEN sample [61]. (**b**) GI4E sample [28]. (**c**) I2HEAD sample [20]. (**d**) MPIIGaze subset sample [56]. (**e**) U2Eyes sample [57].

In 2013, the intelligent behavior understanding group at the Imperial College London re-labeled many stat-of-the-art facial landmark databases with images that are captured under unconstrained conditions (in-the-wild) using the multi-PIE [58] 68 points mark-up. Among these databases are LFPW [59], AFW [60], HELEN [61], 300-W [62], and IBUG [63] databases which together compose a large dataset with 4,437 real-world facial images with accurate labelings. In Figure 2a, a sample from HELEN dataset can be seen. However, the multi-PIE 68 points mark-up does not include pupil center so, for the purpose of this work, a manual labeling procedure has been done by a single annotator with the aim to annotate the pupil centers in these databases. The resulting dataset, named Pupil-PIE (PUPPIE), contains 1791 images with the 2 pupil centers and can be downloaded from https://www.unavarra.es/gi4e/databases/elar (accessed on 4 October 2021). The images have been selected based on whether the pupil annotation can be done accurately, i.e., images in which glasses are worn or in which one eye is hidden by hair have been excluded.

#### 3.1.2. GI4E

GI4E [28] is a database containing images of 103 users gazing at 12 different points on the screen in a standard desktop lab conditions scenario. One of the outstanding characteristics of this database is the accuracy of the labeling procedure. The images contain labels for pupil center and eye corners. Each image has been marked by three independent individuals, and the final label has been calculated as the mean value among the three, assuring highly accurate labels. In Figure 2b, a sample from this database is shown.

#### 3.1.3. I2Head

I2Head [20] is a database combining ground-truth data for head pose, gaze and a simplified user’s head model for 12 individuals. In Figure 2c, a sample from I2Head dataset can be observed. The system is used to register the user’s pose and face images with respect to the camera while gazing different grids of points. For each user, 8 sessions are recorded in static and free head movements scenarios. Among those 8 sessions, 4 recordings were made in a centered location, while in the other 4 the user was asked to translate to extreme positions with respect to the camera. The database provides images, 3D poses, and fixation points but 2D data, i.e., image labels, are not included in the dataset. However, in [64] a manual re-labeled procedure was done. Three individuals participated in the labeling procedure and mean landmarks were obtained for eye corners and pupil centers.

#### 3.1.4. MPIIGaze Subset

MPIIGaze [56] contains 213,659 images collected from 15 participants during natural everyday laptop use over more than three months. This is one of the largest and most varied and challenging datasets in the field. However, some images include the whole face while others do only contain a cropped version of the eye area. Labels for the eyelids are provided in the image together with some 3D information, such as estimated head pose and gaze direction with respect to the camera. The authors claim that a subset containing 10,848 images has been manually annotated including eye corners, pupil centers, and specific facial landmarks. These manually annotated images present acceptable accuracies regarding pupil center and eye corners for several applications, but they cannot be considered suitable for the eye landmark detection task. In order to compensate for this fact and be able to work in more accurate conditions, in [64] a manual re-labeled procedure was done following the same guidelines as the I2Head database. In total, 39 images per user were selected among the 15 subjects included in the original annotation set, resulting in a total of 585 manually annotated images. In Figure 2d, a sample from MPIIGaze database is shown.

#### 3.1.5. U2Eyes

U2Eyes [57] is a binocular database of synthesized images reproducing real gaze tracking scenarios. It was created by duplicating the mesh provided by UnityEyes [65], adding essential eyeball physiology elements and modeling binocular vision dynamics. U2Eyes database includes 1000 users but only 20 users are publicly available. Each user looks at two grids of 15 and 32 points, respectively, with 125 different head poses, resulting in a total of 5875 images per user. Head pose, gaze direction information and 2D/3D landmarks are provided as part of the annotated data. In Figure 2e, a sample from U2Eyes is shown.

### 3.2. Preprocessing

In order to normalize the images, a preprocessing step has been made. First, the eye region is detected. For that purpose, the multi-task cascaded framework based on CNN (MTCNN) proposed by Zhang et al. [66] is used. The facial landmarks estimated by MTCNN are used to create an eye region bounding box. Then, the image is cropped using the eye bounding box. Once the image is cropped, it is resized to a resolution of 128 × 256 pixels and the pixel values of the image are normalized between −1 and 1.

### 3.3. Pupil Center Detection

The method proposed in this work follows a fine-tuning process, i.e., taking a model trained on a database for some task (backbone), adding some layers and tweaking all or some of its weights for another database and task.

**Figure 3:**
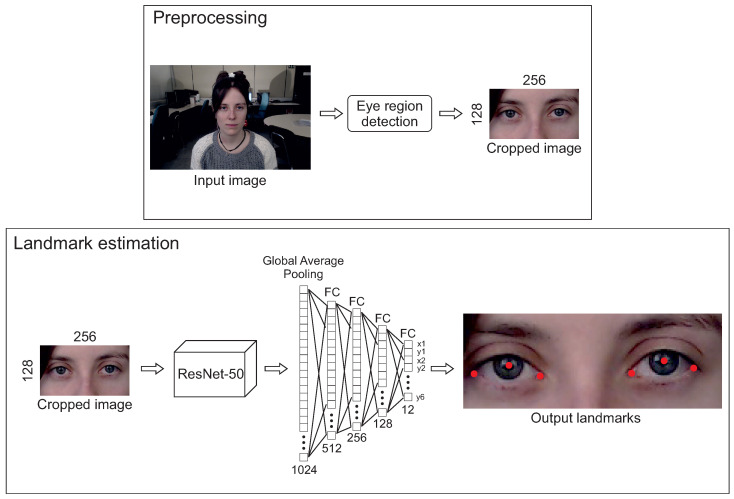
Method proposed. Top: preprocessing step in which the eye region is detected and the image [20] is cropped and resized to a resolution of 128 × 256. Bottom: eye landmark estimation method proposed. The backbone consists of a Resnet-50 followed by a fully connected regression network to obtain the eye landmarks detection.

The architecture of our network is similar to the one used in DeepLabCut [52]. It consists of the ResNet-50 [24] pretrained with ImageNet dataset [67] as backbone, but instead of being followed by specific deconvolutional layers to produce spatial probability densities to each landmark, our network is followed by a global average pooling layer and four fully connected layers with ELU activation function to compute the x and y coordinates of the six eye landmarks (four eye corners and two pupil centers). The architecture proposed can be shown in Figure 3.

The training is divided into two steps, first the backbone weights are frozen and a training of 500 epochs is performed in order to initialize the weights of the fully connected layers. Then, a longer training of 5000 epochs is done with all the weights unfrozen. The optimizer used is Adam with $\beta_{1}=0.9$, $\beta_{2}=0.999$, $\epsilon =1\times 10^{-7}$ and a learning rate of $6\times 10^{-4}$ for the first 500 epochs and $4\times 10^{-5}$ for the next 5000 epochs. In both steps, a batch size of 64 has been used.

In order to enlarge the data and help the model to generalize better, a data augmentation technique has been performed. In training, preprocessed images can be flipped with a probability of 0.5, rotated between −25 and 25 degrees, and scaled with a factor between 0.75 and 1.25.

The problem is approached as a regression task and the loss function is defined as

(1) $$L\mathrm{oss}=\frac{1}{NL}\sum_{i=1}^{N}\frac{\sum_{l=1}^{L}\left|x_{i,l}-{\hat{x}}_{i,l}\right|+\left|y_{i,l}-{\hat{y}}_{i,l}\right|}{IPD_{i}},$$

which corresponds to a $\ell_{1}\;norm$ minimization. Variables $\left(x_{i,l},y_{i,l}\right)$ and $\left({\hat{x}}_{i,l},{\hat{y}}_{i,l}\right)$ correspond to the (x,y) coordinates of the ground-truth and estimated landmark *l* on image *i*, *N* is the total number of images and *L* is the number of landmarks. Each landmark error is normalized by the inter-pupillary distance IPD, calculated as

(2) $$IPD_{i}=\sqrt{{\left(x_{i,pleft}-x_{i,pright}\right)}^{2}+{\left(y_{i,pleft}-y_{i,pright}\right)}^{2}},$$

where subscripts pleft and pright refer to landmark indexes corresponding to left and right pupil centers.

## 4. Evaluation

**Figure 4:**
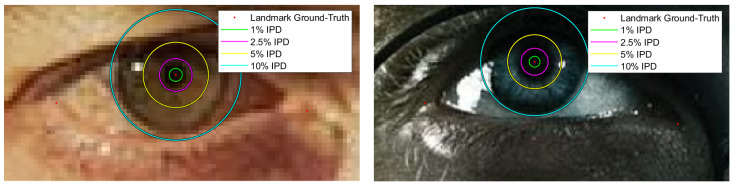
Distances from ground-truth landmark as percentages of inter-pupillary distance. Ground-truth landmarks obtained by the manual labeling procedure explained in Section 3.1.1 are represented by the red points. Green circle represents a distance equivalent to 1% of IPD, magenta a 2.5%, yellow 5%, and cyan 10%.

To evaluate the accuracy of the proposed algorithms and to compare it with state-of-the-art, the relative error measure proposed by Jesorsky et al. [68] has been used. This is formulated by:

(3) $$e_{max}=\frac{max(d_{left},d_{right})}{IPD},$$

where $d_{left}$ and $d_{right}$ are the euclidean distances between ground-truth and estimated left and right pupil centers, and IPD is the inter-pupillary distance in Equation (2). The maximum of $d_{left}$ and $d_{right}$ after normalization is defined as *maximum normalized error* $e_{max}$. The accuracy is calculated as the percentage of images for which this error is below specific thresholds. State-of-the-art methods are usually compared using $e_{max}<0.025\left(2.5\%\right)$, $e_{max}<0.05\left(5\%\right)$ and $e_{max}<0.1\left(10\%\right)$ although some of them do not provide results for accuracy below 5%. Another interesting way to do a performance analysis of the proposed methods that is usually shown in papers, is the cumulative error histogram, which represents the accuracy continuously, i.e., it shows the proportion of images with an error less than each percentage value of IPD. In Figure 4 different distances from ground-truth labels can be shown as percentages values of the inter-pupillary distance, green circle represents a distance equivalent to 1% of IPD, while magenta, yellow and cyan represent the 2.5%, 5%, and 10% of the IPD, respectively. It can be observed that a 10% of the IPD is equivalent to the iris size and a value between 2% and 5% is comparable with the pupil size (depending on the constriction of the pupil).

Regarding the experiments, a model has been trained on PUPPIE dataset and tested on the rest of databases. In order to tune the hyper-parameters presented in Section 3, 80% of PUPPIE database has been used for training, and 20% for validation. The specific details about the tests carried out, i.e., the databases and number of images used in train and test stages and the figures and tables in which results are shown, are summarized in Table 2. It should be noted that, although our method is training-based, our model is trained and tested with completely different databases, which allows us to obtain results over all images of the databases. However, as MTCNN is used to detect the region of the eye, there could be images in which the detection fails. The percentage of images in which MTCNN algorithm has detected the face are 100% for GI4E and I2HEAD datasets, 74.53% for MPIIGaze subset and 68.07% in U2Eyes. This difference in the ratio is due to the fact that, as it could be seen in Figure 2, many images of MPIIGaze dataset and the images of U2Eyes dataset show only the eye region of the face. Therefore, results of our method have also been obtained by generating the eye bounding box from the ground-truth landmarks instead of using the MTCNN face detector. This method in which the generation and cropping of the eye bounding box step has been done using the ground-truth landmarks instead of using the MTCNN detector has been named Ours ground-truth cropped images (Ours GT-CI).

In Table 3, an accuracy comparison on GI4E dataset with state-of-the-art methods is provided. As already said, results are presented as the percentage of images for which the error is below specific thresholds. Some of the state-of-the-art methods do not provide results for accuracy below 5% of IPD. However, an estimate can be made using the cumulative error histogram provided in the papers. These estimations from the graphs are written in italics. Our method achieves an accuracy of 96.68% in the most challenging threshold value, i.e., $e_{max}\leq 0.025$, when MTCNN face detector is used to create the eye bounding box and an accuracy of 98.46% when the eye bounding box is created using ground-truth landmarks. Looking at Figure 4, it can be seen that this means that more than 95% of the images have an error below the size of a constricted pupil. The accuracy for $e_{max}\leq 0.05$ and $e_{max}\leq 0.1$ thresholds is 100%, meaning that every image has a pupil estimation error below the size of a dilated pupil.

The state-of-the-art methods with the most similar results for $e_{max}\leq 0.025$ are Kitazumi18 [48] (96.28%), Choi19 [21] (90.40%), and Levin18 [40] (88.34%), which are already mentioned in the related works section. However, none of them achieve a 100% with $e_{max}\leq 0.05$ and $e_{max}\leq 0.1$.

Regarding the computing time, the preprocessing step using MTCNN takes about 120ms using an Intel Xeon E5-1650 v4 CPU and a Nvidia Titan X (Pascal) GPU and takes about 125ms using an Intel i7-6700k CPU and Nvidia GTX 960 GPU. However, it should be noted that the eye region detection is not the main contribution of this paper, so it is not optimized. It would be possible to use faster detection methods.

Respecting the landmark estimation step, i.e., the main contribution of this paper, our method only takes about 2 ms to estimate pupil center landmarks using an Intel Xeon E5-1650 v4 CPU and a Nvidia Titan X (Pascal) GPU and takes about 5 ms using an Intel i7-6700k CPU and Nvidia GTX 960 GPU. For the same procedure, the method proposed by Choi et al. [21] takes 17 ms using an Intel i7-7700k CPU and Nvidia GTX 1070 GPU. This means that our method achieves an improvement in compute time performance of up to 8 times for the landmark estimation step.

Due to the novelty of the I2HEAD, U2Eyes and the manual re-labeling of the MPIIGaze subset, there are no methods in the literature that report results on these databases. Thus, a comparison between the results obtained for GI4E database and the results obtained for these databases has been made in Table 4. However, as in MPIIGaze subset and U2Eyes database the percentage of images in which MTCNN algorithm has detected the face is less than 100%, results are calculated over the number of detected images instead of the number of total images and the results are marked with an asterisk. As in Table 3, results when the eye bounding box is created using ground-truth landmarks are also shown (Ours GT-CI).

These databases are more challenging than GI4E due to more extreme user head poses, lighting conditions or environment. However, it can be seen that the results achieved on these more challenging databases are better than the ones obtained by state-of-the-art methods on GI4E database.

For the databases in which faces are easily distinguishable and, therefore, face detection works perfectly, i.e., GI4E and I2Head, there are not big differences between the accuracy achieved by generating the eye bounding box using MTCNN face detector and from the ground-truth landmarks. Regarding the databases in which faces are not easily distinguishable because only the eye region of the face is shown, i.e., MPIIGaze subset and U2Eyes, the results achieved using the ground-truth landmarks to generate the eye bounding box over well-detected images are similar than the results achieved on GI4E and I2Head databases.

In the case of U2Eyes synthetic database, results are are worse than the ones obtained on real databases. However, despite the fact that the performance is worse on synthetic databases than on real ones, it still achieves better results than state-of-the-art methods on GI4E database.

**Figure 5:**
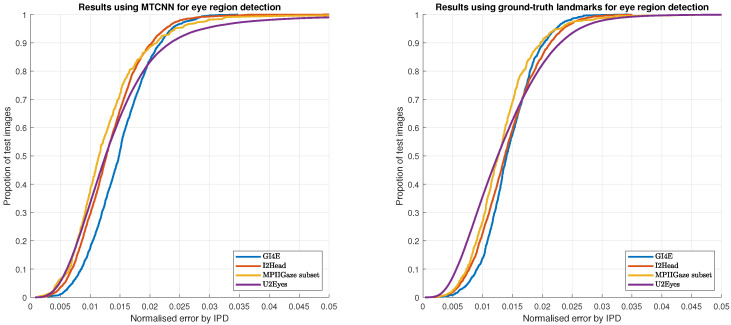
Cumulative error histograms on GI4E, I2Head, MPIIGaze, and U2Eyes datasets using MTCNN (**left**) and ground-truth landmarks (**right**) for eye region detection.

To show the results in a more visual way and to enable future works to obtain accuracies for different thresholds, Figure 5 shows the cumulative error histogram using our method in the aforementioned databases. Comparing the results of initializing the pupil center detection using the MTCNN face detector (left) and the ground-truth landmarks (right), it can be seen the robustness of our method against initializations.

## 5. Conclusions

In this paper, a method for pupil center detection based on convolutional neural networks has been proposed. In order to train the model, a pupil center manual labeling procedure of 1,791 images from an existing facial landmark dataset has been performed and the resulting landmarks annotation is the first contribution of this work. The model has been tested using the well-known GI4E database, outperforming state-of-the-art methods and reducing the computational task time of the landmark estimation step by 3 to 8 times. Furthermore, the model has also been tested using more challenging databases getting outstanding results and enabling a benchmark for future works. The results of our method show how using high quality training data, as well as leading CNN architectures allows to achieve outstanding results with a lower computational time.

## Figures and Tables

**Table 1:** Datasets used in this work. The number of users and images on the original datasets, as well as the number of re-labeled images are summarized.

Database	# of Total Images	# of Selected Images
PUPPIE	4437	1791
GI4E	1236	1236
I2Head	2784	2784
MPIIGaze	10,848	585
U2Eyes	117,500	117,500

**Table 2:** In this table, the databases and the number of images used for train and test are summarized.

Training Dataset		Testing Dataset		Results
PUPPIE	1433 images	GI4E	1236 images	Table 3 and Table 4 & Figure 5
		I2Head	2784 images	Table 4 & Figure 5
		MPIIGaze	585 images	Table 4 & Figure 5
		U2Eyes	117,500 images	Table 4 & Figure 5

**Table 3:** Accuracy comparison for pupil center location on the GI4E database. Estimations from original papers’ graphs are written in italics.

Method	$e_{\max}\leq 0.025$	$e_{\max}\leq 0.05$	$e_{\max}\leq 0.1$
Timm11 [27]	*40.00*	92.40	96.00
Baek13 [69]	*59.00*	79.50	88.00
Villanueva13 [28]	*42.00*	93.90	97.30
Zhang16 [26]	-	97.90	99.60
George16 [30]	*72.00*	89.28	92.30
Gou16 [36]	*72.00*	98.20	99.80
Gou17 [70]	-	94.20	99.10
Levin18 [40]	88.34	99.27	99.92
Larumbe18 [35]	87.67	99.14	99.99
Cai18 [55]	85.7	99.50	-
Xiao18 [31]	*70.00*	97.90	100
Kitazumi18 [48]	96.28	98.62	98.95
Choi19 [21]	90.40	99.60	-
Xia19 [22]	*61.10*	99.10	100
Kim20 [54]	79.50	99.30	99.90
Lee20 [23]	79.50	99.84	99.84
Ours	96.68	100	100
Ours GT-CI	98.46	100	100

**Table 4:** Accuracy comparison for pupil center location on GI4E, I2HEAD, MPIIGaze and U2Eyes databases. Results over MPIIGaze and U2Eyes databases using Ours GT-CI are calculated over the number of detected images and are marked with an asterisk.

	Database	$e_{\max}\leq 0.025$	$e_{\max}\leq 0.05$	$e_{\max}\leq 0.1$
Ours	GI4E	96.68	100	100
	I2Head	97.92	99.96	100
	MPIIGaze	95.18 *	99.54 *	99.77 *
	U2Eyes	91.92 *	98.99 *	99.80 *
Ours GT-CI	GI4E	98.46	100	100
	I2Head	96.88	100	100
	MPIIGaze	97.09	99.83	100
	U2Eyes	93.44	99.93	100

## Data Availability

For more information about the databases, please refer to: http://www.unavarra.es/gi4e (accessed on 4 October 2021) or to the correspondence author.
